# Liver Transcriptomic Profiles of Ruminant Species Fed Spent Hemp Biomass Containing Cannabinoids

**DOI:** 10.3390/genes15070963

**Published:** 2024-07-22

**Authors:** Agung Irawan, Massimo Bionaz

**Affiliations:** 1 Department of Animal and Rangeland Sciences, Oregon State University, Corvallis, OR 97331, USA; irawana@oregonstate.edu; 2 Animal Science Study Program, Universitas Sebelas Maret, Surakarta 57126, Indonesia

**Keywords:** cannabinoids, CBD, liver, ruminant, THC, transcriptomic

## Abstract

The inclusion of spent hemp biomass (SHB), an extracted byproduct from industrial cannabidiol (CBD) production, in the diets of dairy cows and lambs appears to be safe with minor effects on the metabolism, including a decrease in circulating cholesterol and increase bilirubinemia, both associated with liver metabolism. Those effects could be consequence of the presence of cannabinoids, particularly Δ^9^-tetrahydrocannabinol (THC) and CBD in the SHB. This study aimed to study the transcriptional profile of the liver of dairy cows and lambs fed SHB. Dairy cows received SHB or alfalfa pellet for four weeks of intervention (IP) and four weeks of withdrawal periods (WP). Finishing lambs were fed a control diet (CON), 10% (LH2), or 20% (HH2) SHB for 2 months or 1 month followed by 1-month SHB withdrawal (LH1 and HH1, respectively). RNA sequencing was performed, and the mRNA was annotated using the latest reference genomes. The RNAseq data were filtered, normalized for library size and composition, and statistically analyzed by DESeq2. The bioinformatic analysis was performed by using DAVID, Gene Set Enrichment Analysis (GSEA), and the Dynamic Impact Approach. Using a 0.2 FDR cut-off, we identified only ≤24 differentially expressed genes (DEG) in the liver by feeding SHB in dairy cows and a larger number of DEGs in lambs (from 71 in HH1 vs. CON to 552 in LH1 vs. CON). The KEGG analysis demonstrated that feeding SHB in dairy cows and lambs had relatively minor to moderate metabolic alterations in dairy cows and lambs mainly associated with amino acids and lipid metabolism whereas cholesterol synthesis was overall activated in lambs. GSEA identified activation of the PPAR signaling pathway only in dairy cows. We found an opposite effect on activation of metabolism of drug and xenobiotics by cytochrome P450 enzymes in dairy cows and lambs receiving less SHB but an inhibition in HH2 lambs. Immune system-related pathways were inhibited by feeding SHB in lambs, but the impact was minor. Cumulatively, inclusion of SHB containing cannabinoids in dairy and lambs demonstrate very little effects on the alteration of transcriptomic profile of the liver.

## 1. Introduction

Worldwide, hemp (*Cannabis sativa* L.) is an economically important agricultural commodity [1,2,3]. The extraction of cannabidiol (CBD) produces spent hemp biomass (SHB), which could be a valuable feed ingredient for ruminants, owing to its excellent nutritional profile, although it still presents a substantial content of cannabinoids, including ∆^9^-tetrahydrocannabinol (THC) [4]. Our prior studies revealed that feeding SHB has no impact on the production performance and health of finishing lambs and lactating dairy cows [4,5]. However, in the same studies, minor effects on metabolism, liver, immune function, and oxidative stress parameters were observed.

A consistent increase in bilirubinaemia upon feeding SHB was observed in lambs and dairy cows [4,5], indicating a possible decrease in liver clearance capacity [6,7,8]. It is well known that CBD has a strong inhibitory effect on several cytochromes, P450 isozymes, and UDP glucuronosyltransferase [6,7,8], responsible for xenobiotic metabolism and clearance in the liver [6]. Furthermore, our studies [4,5] revealed a reduction of cholesterol concentration in plasma. Those data indicated a possible role of cannabinoids present in the SHB on hepatic lipid metabolism.

Earlier literature suggested that CBD possesses antioxidant and anti-inflammatory properties and is often known to have neuroprotective, cardioprotective, and cytoprotective effects [7,8]. Research in monogastric animals revealed a role of CBD in preventing several liver diseases, likely by the reduction of inflammation, although it remains unclear if CBD acts directly on the liver [9]. Whole transcriptome analysis was performed on a human hepatic cell line (HepG2) treated with CBD, demonstrating a transcriptomic response of the liver to this cannabinoid [10]. An experiment using a mice model revealed that THC-attenuated hepatic inflammation and liver neutrophil-mediated injury by regulating the transcription of genes in the white adipose tissue via activation of peroxisome proliferator-activated receptor γ (*PPARγ*) [11], suggesting a therapeutic potential of THC on alcoholic liver disease. The Δ^9^-THC, on the other hand, is well known to cause numerous adverse effects, such as impairing neuronal systems and mitochondrial function [12,13]. However, interactions may exist between CBD and THC, in line with recent findings, suggesting that CBD might reduce some adverse effects of THC in humans [13].

The effects observed in our prior studies feeding SHB to lambs and dairy cows [4,5] are indicative of a role of cannabinoids on the liver. Based on the evidence provided above, the effect observed may be due to changes in the transcriptome of the liver. This organ is a central hub playing many essential physiological roles, including metabolism and detoxification [14]. Thus, the objective of this study was to investigate the effects of feeding SHB on liver transcriptomic by performing RNA-seq on hepatic tissues collected in lambs and dairy cows in our prior studies [4,5].

## 2. Materials and Methods

### 2.1. In Vivo Study and Experimental Designs

**Figure 1:**
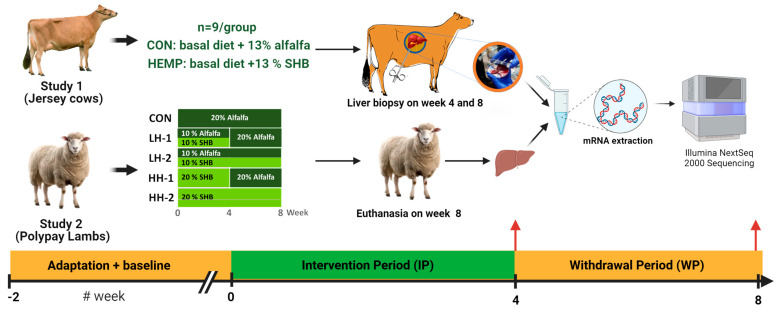
Overview of the experimental design of lactating Jersey cows and finishing Polypay lambs in the spent hemp biomass feeding studies.

The Institutional Animal Care and Use Committee of Oregon State University approved the in vivo studies involving two independent feeding trials using lambs and dairy cows. The studies were conducted to assess if SHB is a suitable feed ingredient for ruminants. In both studies, SHB was used as an alternative to alfalfa meal, each comprising four weeks of the intervention period (IP) and a withdrawal period (WP) (Figure 1). Our previous publications reported details about animal management, experimental designs, and diets, as well as the calculation of the power analysis [4,5]. Briefly, 35 Polypay lambs were randomly separated into individual pens to receive five iso-nitrogenous and iso-caloric diets: control (CON; 7 lambs), 10% SHB (LH; 14 lambs), and 20% SHB (HH; 14 lambs). The animals fed SHB were divided into two equal groups at 4 weeks of the feeding trial (each with 7 lambs) in which the first groups (LH1 and HH1) were fed the CON diet, while the other groups (LH2 and HH2) received SHB in the diets until the end of the experiment (8 weeks total). The second experiment was performed with 18 Jersey late-lactating cows receiving iso-nitrogenous and iso-caloric diets top-dressed with either 13% (as DM) alfalfa pellet (CON) or 13% SHB. The animals received dietary treatments for up to 4 weeks (intervention period or IP), followed by 4 weeks where SHB and alfalfa pellets were withdrawn from the diet (withdrawal period or WP). Information on SHB intake, cannabinoid concentration, and cannabinoid intake has been reported in our previous publications [4,5,15]. Briefly, the dairy cows consumed 1.22 ± 0.55 kg DM/d (7.5% of the diet), corresponding to the ingestion of 74.1 ± 16.3 mg cannabinoids/kg BW with 0.79 ± 0.17 mg Δ^9^-THC/kg BW and 55.2 ± 12.2 mg CBD/kg BW. For the lambs, they consumed between 93.5 (LH1) to 216.7 (HH2) mg cannabinoids/kg BW with between 0.99 and 2.30 mg Δ^9^-THC/kg BW and between 18.2 and 42.1 mg CBD/kg BW (see details in Irawan et al. [15]).

### 2.2. Sample Collections

At the end of the experiment, the lambs were slaughtered at the Clark Meat Science Center at Oregon State University. Liver was removed from the animals after evisceration and approx. 1 g of the left lobe of the liver was dissected using a #10 surgical blade (327-1504, Integra Miltex, York, PA, USA). Biopsy of the liver was performed in dairy cows at the end of the IP and at the end of the WP. To collect the liver tissue, a small incision was made using a #10 surgical blade (327-1504, Integra Miltex, York, PA, USA). A 6 mm i.d. trocar was used to collect approximately ±600 mg liver tissue. In both experiments, the dissected tissue was immediately transferred to a sterile dish (351029, Corning Falcon, Corning, NY, USA) and rinsed using sterile phosphate-buffered saline (25-508P, Genclone, El Cajon, CA, USA) to remove blood contaminant. The tissue was then transferred into a 1.5 mL cryovial (Cat# 10018-760, VWR), flash frozen by immersing the cryovial in a portable liquid N tank, transported to the laboratory, and preserved at −80 °C until transcriptomic analysis.

### 2.3. RNA Extraction, Library Preparation, and Sequencing

Between 30 and 100 mg of liver tissue was transferred into 1.5 mL screw-cap vials (490003-520, VWR, Radnor, PA, USA) prefilled with 3.2 mm bead and 1200 µL of ice-cold TRIzol reagent (15596026, Thermo Scientific, Waltham, MA, USA). The tissue was intermittently disrupted using a bullet blender (Model BBX24, Next Advance Inc., Troy, NY, USA) at high speed (number 9 speed) three times for one minute each, resting for 1 min in ice in between. The three cycles of disruption were sufficient to completely disrupt the tissue in the reagent without any visible piece of tissue. Immediately after disruption, the supernatant was transferred into a 1.7 mL sterile microtube, and 120 μL cold chloroform was added to the tube, mixed, and incubated on ice for 10 min. The samples were centrifuged at 4 °C for 15 min at 15,000× *g*. The upper-phase supernatant was used for RNA purification using Zymo Quick-RNA Miniprep Kit (cat #R1054, Zymo Research, Irvine, CA, USA) following the manufacturer’s instructions. The concentration and purity of isolated RNA were measured using NanoDrop^TM^ Spectrophotometer (Thermo Scientific, Wilmington, NC, USA). The RNA purity was assessed using 260/280 absorbance. The RNA Integrity Number (RIN) was analyzed using an Agilent Bioanalyzer 2100 (G2939BA, Agilent, Santa Clara, CA, USA). Samples with RIN > 7.0 were used for RNA sequencing. The RINs of the samples from lambs and dairy cows were 7.51 ± 0.44 and 7.48 ± 0.28, respectively. The sequencing was performed by the Center for Quantitative Life Sciences (CQLS) at Oregon State University.

The QuantSeq 3’ mRNA-Seq Library Prep Kit FWD for Illumina (#015, Lexogen GmbH, Vienna, Austria) was used for cDNA library construction following the manufacturer’s guidelines. Single-end reads of 100 bp mRNA sequencing were performed at the CQLS using the P2 and P3 flow cells of the Illumina NextSeq 2000 platform (Illumina, San Diego, CA, USA).

### 2.4. Quality Control, Processing, and Alignment of Reads

Quality of raw reads was assessed using MultiQC v1.8 (https://multiqc.info/ (accessed on 20 January 2024)). Adapters and low-quality reads based on PHRED score (Q < 30) were trimmed and filtered using the BBDuk program (https://github.com/BioInfoTools/BBMap (accessed on 20 January 2024)) using default parameters. The latest genome reference index of *Bos taurus* (BT; GCF 002263795.2 ARS-UCD1.3) and *Ovis aries* (OA; GCF 016772045.1 ARS-UI-Ramb v2.0), along with the gene transfer format files, were downloaded from NCBI. Gene and transcript alignments to the reference genome were perfomed using STAR v. 2.7.11a (https://github.com/alexdobin/STAR (accessed on 20 January 2024)) [16]. Stringtie v2.2.0 (https://ccb.jhu.edu/software/stringtie/ (accessed 20 January 2024)) was used to assemble, annotate, and generate the genes and transcripts counts. Raw read count matrix of genes and transcript abundance were generated using a phyton bash script provided by the bioinformatics team of CQLS at Oregon State University.

### 2.5. Differential Gene Expression Analysis

Statistical analysis was performed using DESeq2 pipeline [17] in R (RStudio version +463; R Core Team, 2023). The raw reads filtering was applied before the DESeq2 analysis by trimming the genes with ≤3 raw counts, including removing ribosomal RNA that were identified in all of the samples. To be eligible for the DESeq2 analysis, we used a criterion of at least three biological samples of either treatment group having ≥3 raw reads count. The pre-filtering aims to minimize the pipeline bias such as between-group-imbalance filtering that might influence the number of genes passed for the analysis. Then, outliers were assessed with principal component analysis (PCA) by using a regularized log transformation function in the DESeq2 package and Uniform manifold approximation and projection (UMAP) clustering in the RNAchef online tool (https://imeg-ku.shinyapps.io/RNAseqChef/ (accessed on 20 January 2024)) [18]. The outliers’ assessments led to removing two samples in the dairy cows during IP and WP, one sample of CON group and one sample of HH2 group of the lambs’ study. The cut-off criteria for detection and the statistical analysis to assess the differentially expressed genes (i.e., transcripts; DEG) were performed separately for each treatment group vs. the control group. Transcripts were considered DEGs when *p* < 0.05 and FDR-adjusted *p*-value was < 0.20 according to Benjamini-Hochberg. A Venn diagram was created to identify overlapped DEGs between groups using an online tool (https://bioinformatics.psb.ugent.be/webtools/Venn/; accessed on 7 April 2024).

### 2.6. Bioinformatic Analyses

Multiple bioinformatic approaches were used to examine the functional roles of DEGs, including overrepresentation analysis of gene ontology (GO) terms using DAVID functional annotation tool [19], Gene Set Enrichment Analysis (GSEA) using ClusterProfiler [20], and the Dynamic Impact Approach (DIA) [21]. The Kyoto Encyclopedia of Genes and Genomes (KEGG) pathways were analyzed using GSEA and DIA approaches. For DAVID and DIA, all detected genes following the above-described cut-off for each specific comparison were used as a background [22,23].

For DAVID analysis, the upregulated and downregulated DEGs were submitted separately to identify the activation or inhibition of the GO terms output. The functional annotation of biological process (BP), cellular component (CC), and molecular function (MF) was determined using the thresholds option of minimum 3 genes in the term at *p* < 0.05. The GO terms obtained from upregulated genes were presented as positive fold-enrichment value and GO terms from downregulated genes were presented as negative fold-enrichment value in the figures. The overlapped DEGs identified between LH2 and HH2 groups were also analyzed using DAVID.

The GSEA was performed using clusterProfiler 4.0 in RStudio [24]. Different from GO enrichment analysis, GSEA uses all transcripts that were detected (i.e., >3 reads in at least 3 samples per group) ranked according to the log2-fold changes data as input [20] to identify top perturbed pathways. The impacted pathways of interests were visualized using Pathview (https://pathview.uncc.edu/ (accessed on 6 April 2024)), which is embedded in the clusterProfiler package in R. Due to the unavailability of a lamb or sheep annotation database, the gene symbols of lambs were firstly converted into BT ENTREZ ID using dbOrtho conversion tools of biological DataBase network (BioDBnet; https://biodbnet-abcc.ncifcrf.gov/db/dbOrtho.php (accessed on 6 April 2024)) before performing the GSEA and DIA functional enrichment analyses.

## 3. Results

### 3.1. Differential Gene Expression Analysis

The mRNA sequencing from the liver samples yielded 11,823,318 ± 1,988,996 and 15,748,863 ± 2,802,552 clean reads per sample for dairy cows and lambs, respectively (Supplementary File S1). After the removal of zero and low-count raw reads, 14,126 and 14,603 annotated genes in IP and WP and 15,842–16,013 annotated genes for each comparison of the liver in lambs were detected, which were included in the subsequent DESeq2 analysis.

**Figure 2:**
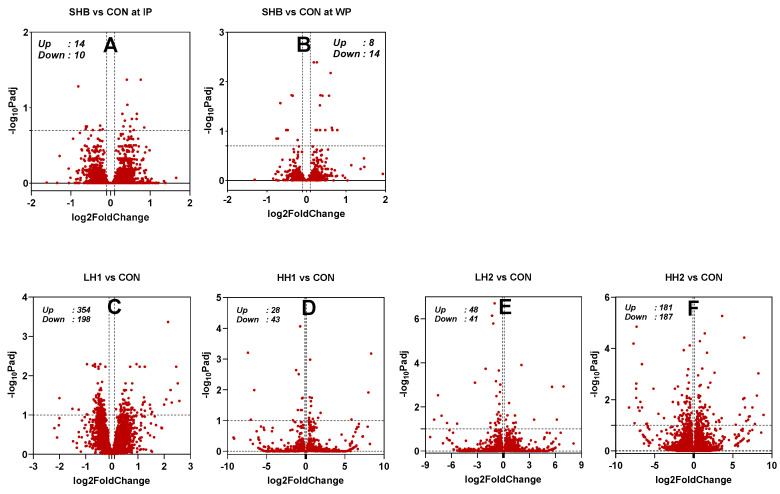
Volcano plots of differentially expressed coding protein transcripts between dairy cows fed spent hemp biomass (SHB) and control diet (CON) during the intervention period (IP, (**A**)) and withdrawal period (WP, (**B**)) and lambs fed SHB for 4 weeks + 4 weeks of withdrawal period [10% SHB (LH1, (**C**)) and 20% SHB (HH1, (**D**))] and lambs fed for 8 weeks with SHB [10% SHB (LH2, (**E**)) and 20% SHB (HH2, (**F**))].

**Figure 3:**
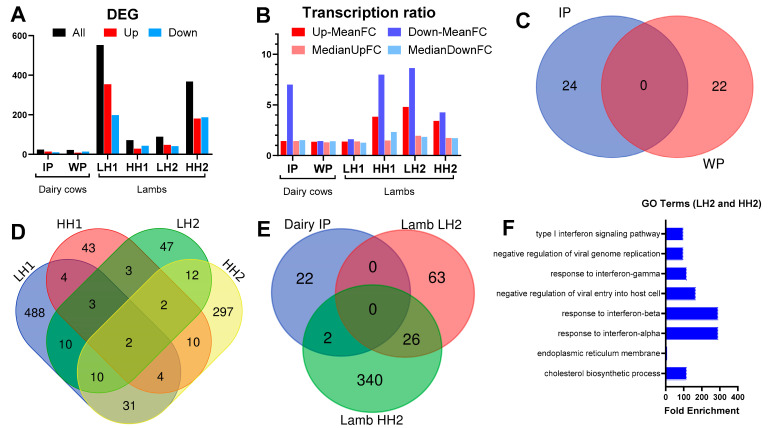
The number (**A**) and expression ratio ((**B**), both as mean and median) of differentially expressed genes (DEGs) for each comparison. (**C**) Venn diagram of the overlapped DEGs between SHB vs. CON during the intervention (IP) or withdrawal (WP) period in dairy cows. (**D**) Venn diagram of the overlap DEGs between the various comparisons in the lamb study (see caption of Figure 2 for the groups). (**E**) Venn diagram of overlap DEGs in the dairy study (HSHB vs. CON during the IP) and the study with lambs. (**F**) Gene ontology (GO) terms enriched (*p*-value < 0.05) of the 26 overlap DEGs between LH2 and HH2 groups.

*Dairy cows*: The PCA (Supplementary File S2, Figure S1) reveals a low separation of the groups by the first and second principal components (PC1 and PC2), which only explains 17% and 11% variance. The statistical results are available in Supplementary File S3. During the IP, feeding SHB resulted in 24 DEGs (14 upregulated and 10 downregulated; Figure 2 and Figure 3A). Following SHB withdrawal, 22 DEGs were identified (14 upregulated and 8 downregulated; Supplementary File S3, Figure 2 and Figure 3A). There was a similar fold change between cows receiving SHB and CON in IP than WP (Figure 3B). There was no overlapping DEG of SHB vs. CON between IP and WP (Figure 3C).

*Lambs*: There was a high within-group variance, with PC1 and PC2 explaining only 10% and 8% of the variance, respectively (Supplementary File S2, Figure S1). As shown in the volcano plots (Figure 2) and the number of DEGs for each comparison in Figure 3A, the number of DEGs identified in the lamb study was larger compared to the dairy cows’ study. The largest number of DEGs was identified in the LH1 vs. CON (552 DEGs; 354 upregulated and 198 downregulated) followed by HH2 vs. CON comparison (368 DEGs; 187 upregulated and 181 downregulated). In LH2 vs. CON, less than half of the number of DEGs was found compared to HH2 vs. CON. Despite a larger number of DEGs, the LH1 vs. CON had a lower mean fold change in the DEGs compared to LH2 vs. CON or HH2 vs. CON (Figure 3B). Complete results of the DESeq2 in the study with lambs are available in Supplementary File S4. There were few common DEGs between the various comparisons, with the largest overlap between LH1 and HH2 vs. CON (47 DEGs) followed by LH2 and HH2 vs. CON with 26 common DEGs (Figure 3D). We only detected two DEGs that were commonly affected by feeding SHB in lambs and dairy cows (HH2 vs. CON in lamb and SHB vs. CON during the IP in dairy cows, Figure 3E): *QPRT* that was up-regulated by feeding SHB in both species, and *GSTM3* that was upregulated in dairy cows and down-regulated in lambs.

### 3.2. Functional Analysis of Differentially Expressed Genes

*Dairy cows*: No significantly enriched GO terms were identified during the IP. However, the GSEA identified activation of ‘potassium ion transmembrane transport’ and suppression of several terms related to DNA binding and regulation and ‘cholesterol binding’ in the dairy cows fed SHB vs. CON during IP (Supplementary File S2, Figure S2). Meanwhile, enrichment of several functional GO terms in the SHB vs. CON during WP were identified in up-regulated DEGs using DAVID, including ‘acute-phase response’, ‘proteolysis’, and ‘extracellular space’ (Supplementary File S5).

**Figure 4:**
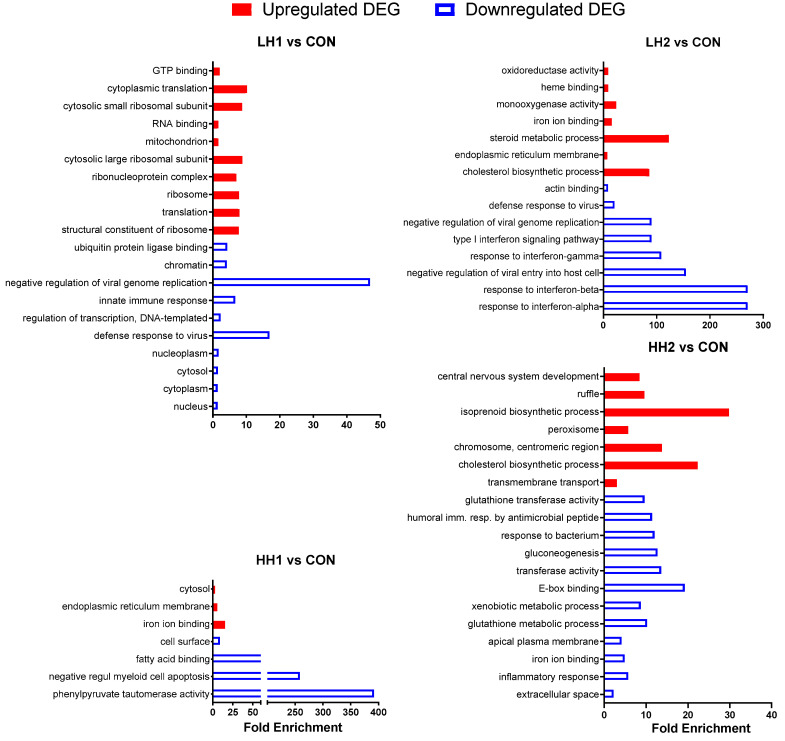
DAVID Gene Ontology analysis of differentially expressed genes in lambs fed SHB for 4 weeks + 4 weeks of withdrawal period [10% SHB (LH1) and 20% SHB (HH1)] and lambs fed for 8 weeks with SHB [10% SHB (LH2) and 20% SHB (HH2)]. The X-axis provides fold enrichment.

*Lambs*: Main GO functional annotations are shown in Figure 4, and the full GO results are available in the Supplementary File S6. Several GO terms were enriched toward negative pattern in LH1, LH2, and HH2 vs. CON, including terms related to the immune response. The cholesterol biosynthetic process was highly enriched among up-regulated DEGs in LH2 and HH2 vs. CON (Figure 4). The activation of the cholesterol biosynthetic process was also identified in the GSEA for LH2 and HH2 vs. CON (Supplementary File S6). The down-regulated DEGs in HH2 vs. CON were also enriched with genes associated with xenobiotic, gluconeogenesis, xenobiotic, glutathione, and inflammatory processes (Figure 4). The up-regulated DEGs in LH1 vs. CON were enriched with terms related to ribosomes and protein synthesis (Figure 4). In contrast to LH2 vs. CON, iron ion binding was inhibited in HH2 vs. CON.

### 3.3. KEGG Pathways Analysis

*Dairy cows*: KEGG pathway analysis was performed using GSEA and DIA approaches (Supplementary File S2, Figures S3–S5). Consistent among DIA and GSEA was the induction of ‘drug metabolism–cytochrome P450′ and ‘xenobiotic metabolism by cytochrome P450′ pathways, which were among the top 10 impacted pathways in SHB vs. CON during IP (Supplementary File S7). Activation of ‘PPAR signaling pathway’, ‘proteosome’, and ‘spliceosome’ were also identified through GSEA in the same comparison but not DIA (Supplementary File S7; Supplementary File S2, Figures S3 and S4). The DIA analysis revealed an overall minor impact on KEGG pathways with ‘metabolism’, especially the ‘metabolism of cofactors and vitamins’, which was the most activated pathway (Supplementary File S2, Figure S4). During WP, the DIA analysis of the transcriptomic profile of dairy cows fed SHB during the IP revealed suppression of ‘lipid metabolism’ and ‘metabolism of terpenoids and polyketides’ subcategories (Supplementary File S2, Figure S4). In particular, during the WP, fatty acid and terpenoid backbone biosynthesis were the most inhibited pathways in cows fed SHB during the IP (Supplementary File S2, Figure S5 and Supplementary File S7). Another noticeable effect revealed by GSEA analysis was the activation of immune-related pathways and inhibition of NF-kappa B signaling and cell cycle pathways in cows that received the SHB during the IP period (Supplementary File S2, Figure S4).

**Figure 5:**
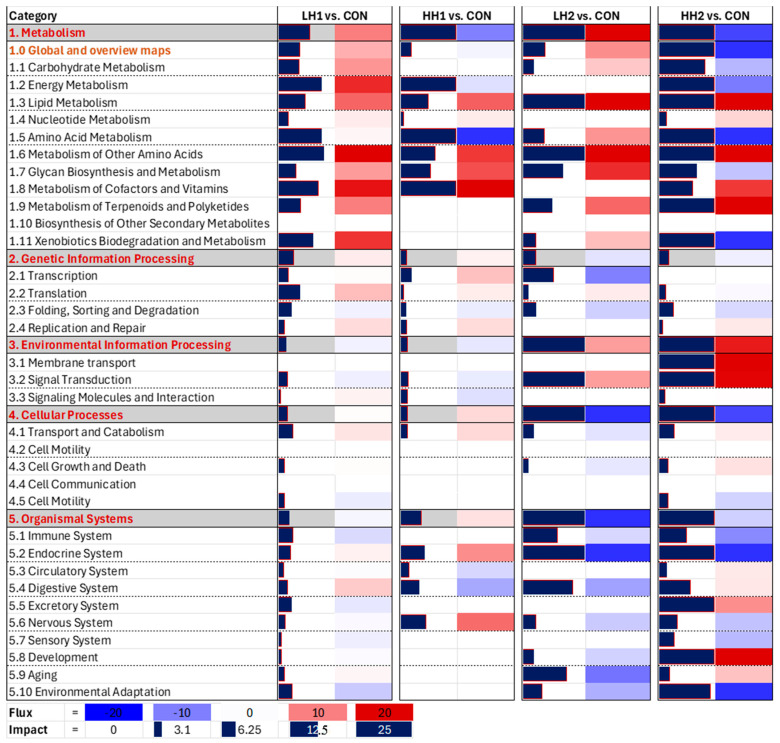
Dynamic Impact Approach summary of KEGG pathways of the liver transcriptomic of lambs fed SHB for 4 weeks + 4 weeks of withdrawal period [10% SHB (LH1) and 20% SHB (HH1)] and lambs fed for 8 weeks with SHB [10% SHB (LH2) and 20% SHB (HH2)].

*Lambs*: DIA results revealed that the induction of KEGG pathways due to dietary SHB in lambs was greater on the LH2 and HH2 vs. CON than the LH1 and HH1 vs. CON (Figure 5). According to DIA, HH2 treatment induced metabolism and cellular processes. In the metabolism category, the highest induction was observed on lipid metabolism (Figure 5), particularly due to high activation of ‘steroid biosynthesis’ (Supplementary File S8), while the cellular process category was inhibited due to high inhibition of the ‘Signaling pathways regulating pluripotency of stem cells’ (Supplementary File S8). The most noticeable effect was the large inhibition of carbohydrate, amino acid, energy, and xenobiotic metabolism in HH2 vs. CON; the same categories of pathways were instead minimally induced or inhibited in LH2 vs. CON (Figure 5). Pathways involving amino acids included Ala, Asp, Glu, Arg, Tyr, Phe, Trp, and sulfur-containing amino acids (Cys and Met), all inhibited in HH2 vs. CON (Supplementary File S8). Feeding SHB for up to 8 weeks also led to a substantial inhibition of the endocrine system, particularly ‘Melanogenesis’, and moderate inhibition of the immune system, including a common inhibition of the ‘IL-17 signaling pathway’ in both LH2 and HH2 groups (Supplementary File S8). There was a good overlap among the most impacted pathways in LH2 and HH2 groups where ‘steroid biosynthesis’ and pathways related to signal transduction subcategory were activated (Supplementary File S2, Figure S5 and Supplementary File S8). The enrichment analysis results using GSEA revealed the similar finding of activation of steroid biosynthesis pathway in all groups (Supplementary File S2, Figure S3). The effects of SHB withdrawal from the diets of lambs can be observed from the lower overall impacts on KEGG pathways of LH1 and HH1 groups (Figure 5 and Supplementary File S2, Figure S5).

## 4. Discussion

Hemp contains more than 500 secondary compounds [25,26]. Once extracted, some of those compounds are present below the detection limits, as observed for terpenes in our SHB, except for cannabinoids, which are still present in a relatively abundant concentration (ref). Although other secondary compounds can affect the transcriptome, our discussion will focus more on cannabinoids, as those are highly bioactive and abundantly present in the SHB.

The endocannabinoid (EC), system plays a central role in regulating energy homeostasis and metabolism in mammals. In dairy cows, it has been suggested that nutritional intervention targeting EC receptors might be advantageous to increase feed intake, productivity, and health by enhancing lipogenesis and adipogenesis and attenuating stress-induced suppression of dry matter intake [27,28]. CBD in the SHB might be a promising anti-inflammatory compound [29]. The beneficial effects of CBD can be associated with its affinity to bind a series of EC receptors such as CB1, CB2, transient receptor potential vanilloid (*TRPV*), *GPR55*, and *PPARγ* [30]. Thus, activating cannabinoid receptors via phytocannabinoids might be a relevant strategy to improve energy homeostasis in ruminants. A plethora of studies using animal models have demonstrated that THC and CBD are capable of interacting with EC receptors, particularly CB1 (*CNR1*) and CB2 (*CNR2*) within the body [22,31,32,33,34].

To our knowledge, this is the first study evaluating the effect of feeding SHB on the liver transcriptional profile of dairy cows and lambs. We did not detect any transcription of *CNR1* in the liver of dairy cows, and only 12% of the samples in lambs had a detectable *CNR1*. In dairy cows, 30% of the samples and in lambs, 50% of the samples had a detectable *CNR2* transcript; however, in lambs, *TRPV2* and *TRPM8*, two of the known receptors binding cannabinoids [35] were highly expressed in all samples but were not or not as expressed in cows (Supplementary File S1). The low detection of *CNR1* and *CNR2* in the liver is not surprising, given that those genes have usually a low transcription in the liver under normal physiological conditions [36]. In monogastric animals, upregulation of *CNR1* in the liver was reported to be associated with liver diseases such as hepatitis, non-alcoholic fatty liver, and immune dysfunction [37,38]; none of those were observed in our experiments with dairy cows and lambs [4,5]. In those experiments, we instead observed during the first month of feeding SHB to lambs a decreased concentration in the blood of glucose, cholesterol, and paraoxonase, an increase in bilirubin and alkaline phosphatase (ALP), and an increase in urea, bilirubin, β-hydroxybutyrate, and ALP during the second month of feeding SHB. All plasma parameters data indicate an effect on the liver, although the effect on ALP might be associated with bone metabolism [4]. The latter is somewhat confirmed by a lack of change in transcription by feeding SHB in our studies (Supplementary Files S3 and S4). In dairy cows, among blood parameters associated with the liver, we only observed a decrease in cholesterol and an increase in bilirubin while feeding SHB [5]. Thus, we performed the transcriptomic analysis to figure out if the phenotype observed in the blood parameters was due to an effect of feeding SHB on the liver transcriptome.

### 4.1. Most Cannabinoid Receptors Are Virtually Not Expressed in Liver

Transcription of *CNR1* was virtually not present, and *CNR2* was transcribed in only 30 or 50% of the samples for cows and lambs, respectively (Supplementary File S1). Most other receptors that can bind cannabinoids were not transcribed in the liver, except for *TRPV2* and *TROM8*, which were transcribed in all lamb samples but only in a few bovine samples (Supplementary File S1). *PPARγ* is also a very important target of the cannabinoids [11,12]. The transcription of *PPARG* was undetectable for most of the liver samples, or if expressed, the transcription was extremely low (Supplementary File S1). Nevertheless, cannabinoids might have some effects on CB receptors of other tissues/cells within the body, which was not investigated in our study. As it has been well recognized, *CNR1* is abundantly expressed in the nervous system and other peripheral tissues [39] while *CNR2* is expressed primarily within the immune cells [40].

### 4.2. Feeding SHB Has a Minor Effect on the Liver Transcriptome

There is a paucity of data on the transcriptome effect of cannabinoids. An effect on the transcriptome by Δ^9^-THC and CBD has been observed in zebrafish embryos through modulation of cannabinoid receptors and *PPARγ* [31]. An agonistic role of Δ^9^-THC on PPARγ has also been observed in the white adipose tissue of mice [11]. The Δ^9^-THC affects the transcription of several genes in the hippocampal neurons of mice [41], in several cells of the immune system in humans [42], and in bull sperm [43]. Exposure to large doses (615 mg/kg BW) of CBD affects the transcription of >50 genes related to oxidative stress, lipid metabolism, and detoxification in the liver of mice [44].

Our data revealed a minor effect of SHB on the liver transcriptome. Even with a liberal FDR of 0.2, we detected a very low number of DEGs, especially in dairy cows. This is likely due to the lack of adequate expression of the various cannabinoid receptors in this organ, as discussed above. However, the number and/or fold change in DEGs appeared proportional to the amount of SHB in the diet. Dairy cows ate only 7.5% of SHB in the diet, lower than LH2 (10%) and HH2 (20%). As the various cannabinoid receptors had no or low expression in the liver, the effect observed on the transcriptome is likely due to other non-cannabinoid compounds in the SHB. This appears to contrast with data in monogastric animals, where an effect of CBD on the liver, including transcripts, has been reported [9,10].

### 4.3. Feeding SHB Affects Genes Related to Amino Acids and Steroid Synthesis

The KEGG analysis revealed that feeding SHB leads to minor to moderate metabolic alterations in dairy cows and lambs, mainly associated with amino acids and lipid metabolism. In lambs, HH2 group had lower DMI in the first 4 weeks of the feeding period but no significant difference at 8 weeks compared to CON, with both groups having similar growth performance in both periods [4].

Our study revealed an overall activation of the cholesterol biosynthetic process via the GSEA tool in all comparisons in lambs but not dairy cows. Interestingly, cholesterol in blood was somewhat consistently reduced by SHB feeding in dairy cows and lambs, albeit only during the first month of feeding SHB in lambs [4,5]. In lambs, on the other hand, the cholesterol concentration of LH2 and HH2 groups was numerically higher than the first month, which might partly explain the indicated activation of cholesterol synthesis in our transcriptomic analysis in lambs. Thus, it is unlikely that in our study the effect observed in circulating cholesterol is explained by a decrease in transcription of genes coding for the enzymes involved in cholesterol synthesis [5]. As previously argued, the reduced circulating cholesterol is likely due to decreased feed intake [4,5].

### 4.4. Spent Hemp Biomass Does Not Induce Inflammation

The impact of cannabinoids on the immune system has been well established. Among many cannabinoids, THC, CBD, and CBG are compounds of interest; they can partially bind to CB1 and CB2 receptors. In this context, these cannabinoids might activate the CB2 receptor and impact the immune system, as the CB2 receptor is mainly expressed in the immune cells [40]. There is evidence that THC + CBD coadministration exhibited positive effects on immunity [45]. Although in different amounts, the SHB used in our experiment contained both CBD and THC [4].

In dairy cows, endocannabinoid biomarkers in blood are elevated during the peripartum and heat stress indicating a role of this system during major metabolic adaptations, where increase in inflammation, lipolysis, and BW losses are also observed [46]. CBD, which is highly enriched in the SHB used in our experiments [4], is known to have potent anti-inflammatory roles in monogastric animals [29,47]. In dairy cows, we detected an increase in circulating ceruloplasmin while feeding SHB, possibly indicating an increase in inflammation [5]. However, no differences were observed for any other inflammatory parameters, such as the positive acute phase protein haptoglobin and the negative acute phase protein albumin, suggesting the increase in ceruloplasmin was due to other causes, such as a higher level of copper in the diet [5]. To confirm the lack of any inflammation, the transcript for ceruloplasmin (*CP*), haptoglobin (*HP*), albumin (*ALB*), and the various serum amyloid A isoforms (*SAA1*, *SAA2*, and *SAA3*) were not significantly affected by feeding SHB during the IP (Supplementary File S3). Furthermore, we did not detect any enrichment or impact on the inflammatory-related GO terms or pathways.

Blood parameters did not indicate inflammation in lambs fed the SHB [4]. In support of this, the transcriptomic data suggested that the pro-inflammatory IL-17 signaling pathway was inhibited in all treatment groups, even after removing SHB from the diet. This pathway regulates the response to infections with the NFκB proinflammatory regulator as downstream targets controlling the transcription of various interleukins, such as IL6 and IL1 [48]. However, the bioinformatic analysis of the transcriptomic data indicated no large effect on the NFκB pathway (except GSEA indicating an inhibition of this pathway in all groups in lambs, except HH2, see Supplementary File S6) without change in transcription of any interleukin (Supplementary File S4). The bioinformatic analysis of our transcriptomic data only revealed a few terms related to the immune response in lambs receiving the SHB, mostly inhibited, and this was evident in the results of the DIA analysis, with a larger inhibition of those pathways in HH2 vs. CON than LH2 vs. CON (e.g., ‘Complement and coagulation cascades’) (Supplementary File S8). The liver is considered part of the innate immune response system due to the presence of many immune cells [49]. Thus, our data indicate that SHB did not activate the hepatic immune response system and might have decreased inflammation; this could benefit the animals long-term [49].

### 4.5. Data Do Not Support an Effect of SHB on PPAR Signaling Pathway

The GSEA tool revealed an enrichment of the *PPAR* signaling pathway in the liver by feeding SHB to dairy cows; however, the other bioinformatic tools did not confirm this. As displayed in Supplementary File 2, Figure S7, feeding SHB had an overall activation of the PPAR pathway, especially for *PPARα* and *PPARγ*. These *PPAR* isotypes are known to regulate lipid metabolism-related genes and are activated by fatty acids [50,51,52]; however, CBD and other cannabinoids are also activators of those *PPAR*s [53,54]. The activation of *PPAR* by THC and CBD was revealed in a transcriptomic study of zebrafish [31]. The same pathway was not affected in the lambs, where more SHB was fed than cows. Thus, due to the lack of enrichment of PPAR-related pathways in lambs and the indication of the importance of this pathway only by one of the bioinformatic tools, we conclude that the data do not support a strong effect of SHB on the PPAR signaling pathway in the liver of dairy cows and lambs.

### 4.6. Activation of Cytochrome P450 Pathway by SHB

In our studies where SHB was fed to dairy cows and lambs, we observed an increase in circulating bilirubin, suggesting a decreased clearance by the liver, including xenobiotic clearance [4,5]. The CBD and THC are competitive inhibitors of the P450 enzymes [53,54,55,56]. Interestingly, the bioinformatic analysis of our transcriptomic data indicated an overall activation of the ‘drug metabolism–cytochrome P450 pathway’ and ‘Metabolism of xenobiotics by cytochrome P450′ pathways in both dairy cows receiving SHB in both DIA (Supplementary File S2, Figure S5) and GSEA (Supplementary File S2, Figures S3, S8 and S9) and lambs receiving the highest dose of SHB in our study, i.e., HH2 group (revealed only by DIA, Supplementary File S7).

It is broadly known that there are multiple P450 enzymes responsible for THC and CBD metabolism, whereas *CYP2C9* is involved primarily in the THC hydroxylation to 11-hydroxy-D9-tetrahydrocannabinol (active metabolite) and 11-nor-9-carboxy-D9-tetrahydrocannabinol (THC-COOH; inactive metabolite), while *CYP2C19* and *CYP3A4* are responsible for CBD metabolism [57,58,59,60]. None of those previously reported enzymes were affected in our transcriptomic study (Supplementary Files S3 and S4). Thus, the above observation, together with the apparent activation of pathways involved in the xenobiotic metabolism despite the increase in circulating bilirubin, suggesting a decrease in xenobiotic clearance, lend us to propose that a competitive inhibition by cannabinoids was the cause of the reduced liver clearance and not a decrease in expression of P450 enzymes.

## 5. Conclusions

Our data revealed that feeding SHB has a minor effect on the liver transcriptome; this was more evident in dairy cows than lambs, likely due to the difference in the amount of ingested SHB and the duration of the treatment. The minor effect on the transcriptome was likely due to the zero or low transcription of major endocannabinoid receptors in the liver.

Despite the lack of effect on the liver transcriptome, we cannot exclude a transcriptomic effect of cannabinoids on other tissues, such as adipose tissue and the brain. Thus, the physiological effects of feeding SHB observed in our prior studies with lambs and dairy cows could be a consequence of the effect of cannabinoids in those other tissues. Overall, and in line with our prior findings, the data support the safety of SHB as a feed ingredient for dairy cows and lambs. 

## Data Availability

Raw sequencing data have been deposited to NCBI SRA (accession number: PRJNA1050772).

## Supplementary Materials

The following supporting information can be downloaded at: https://www.mdpi.com/article/10.3390/genes15070963/s1.
