# Supplementary material for: Liver Transcriptomic Profiles of Ruminant Species Fed Spent Hemp Biomass Containing Cannabinoids
Source: Genes (Basel). 2024 Jul 22;15(7):963. doi: 10.3390/genes15070963 (PMC11275923; doi:10.3390/genes15070963)
Supplement: Supplementary file 1 [file genes-15-00963-s001.zip › Supplementary File S2.pdf]

Supplementary Table

Table S1. Concentration of cannabinoids in the spent hemp biomass used in the experimental diet.

**Cannabinoids**	**% (Biomass)**	**Proportion (% total cannabinoids)**
CBC	0.070	2.3
CBC-A	0.267	8.9
CBC-Total	0.304	10.1
CBD	0.585	19.4
CBD-A	1.88	62.5
CBD-Total	2.24	74.3
CBDV	<LOQ[1]	
CBDV-A	0.0298*	0.99
CBDV-Total	<LOQ	
CBG	<LOQ	
CBG-A	0.104	3.4
CBG-Total	0.091	3.0
CBL	<LOQ	
CBN	<LOQ	
Δ^8-THC	<LOQ	
Δ^9-THC	0.032	1.1
THC-A	0.040	1.3
THC-Total	0.067	2.2
THCV	<LOQ	
THCV-A	<LOQ	
THCV-Total	<LOQ	
Total Cannabinoids	3.01	100.0

[1]LOQ = limit of quantitation that was 0.0292% for all cannabinoids according to Columbia Laboratories (Portland, OR).

*CBDVA was detected in one sample (out of two samples) just barely above the LOQ

Table S2. Intake of cannabinoids on dairy cows fed spent hemp biomass during feeding intervention period

Items	Concentration (%)	Intake (mg/kg BW/d)
SHB intake, kg/d		1.20±0.24
Cannabinoids	2.98	72.2±15.42
Δ^9-THC	0.03	0.77±0.16
THCA	0.04	0.96±0.20
CBGA[1]	0.10	2.50±0.53
CBD	0.58	14.0±3.00
CBDA	1.88	45.1±9.63
CBC[2]	0.07	1.68±0.36
CBCA	0.27	6.41±1.37

SHB = spent hemp biomass; CBC = cannabichromene; CBCA = Cannabichromenic acid; CBGA = cannabigerolic acid; CBD = cannabidiol; CBDA = cannabidiolic acid; Δ^9-THC = Δ^9-tetrahydrocannabinol; Δ^9-THCA = tetrahydrocannabinolic acid

Supplementary Figures

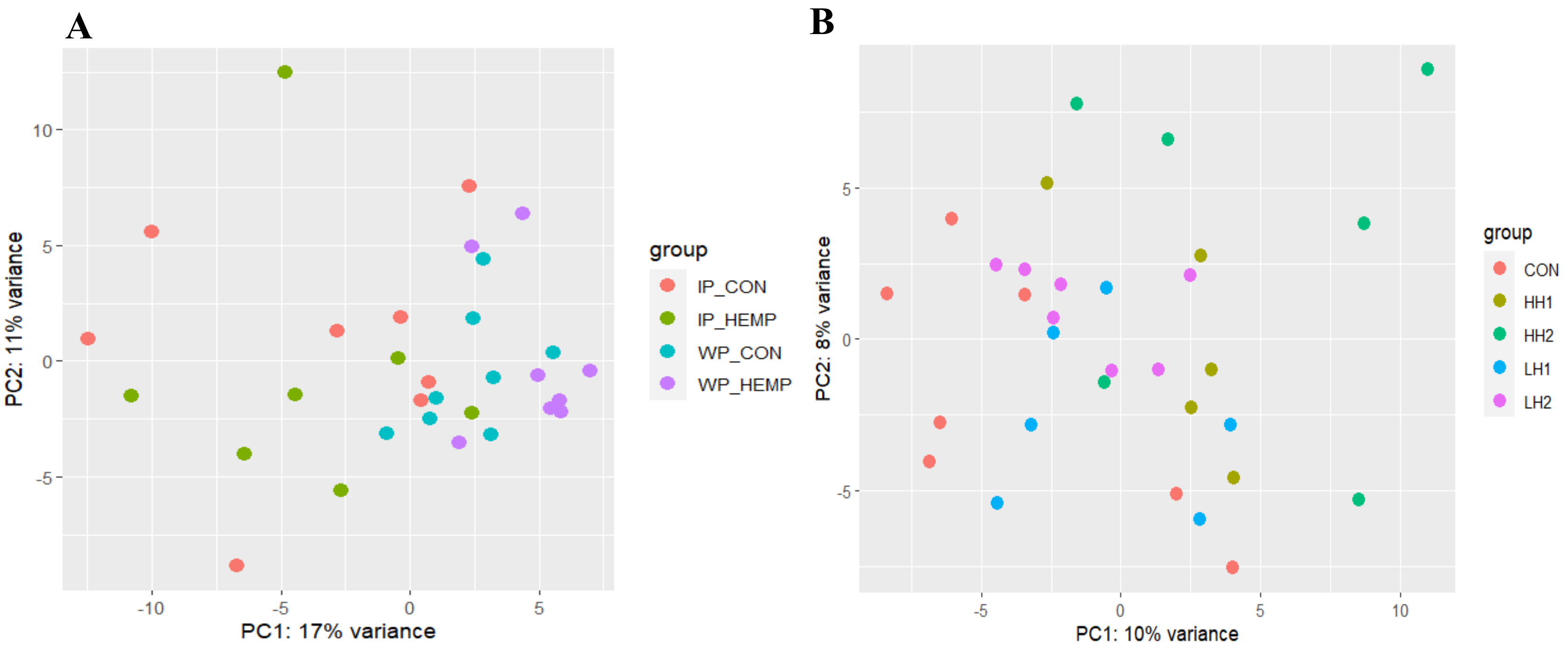

Figure S1. PCA plot of normalized value of gene expression matrix from dairy cows (A) and lambs (B)

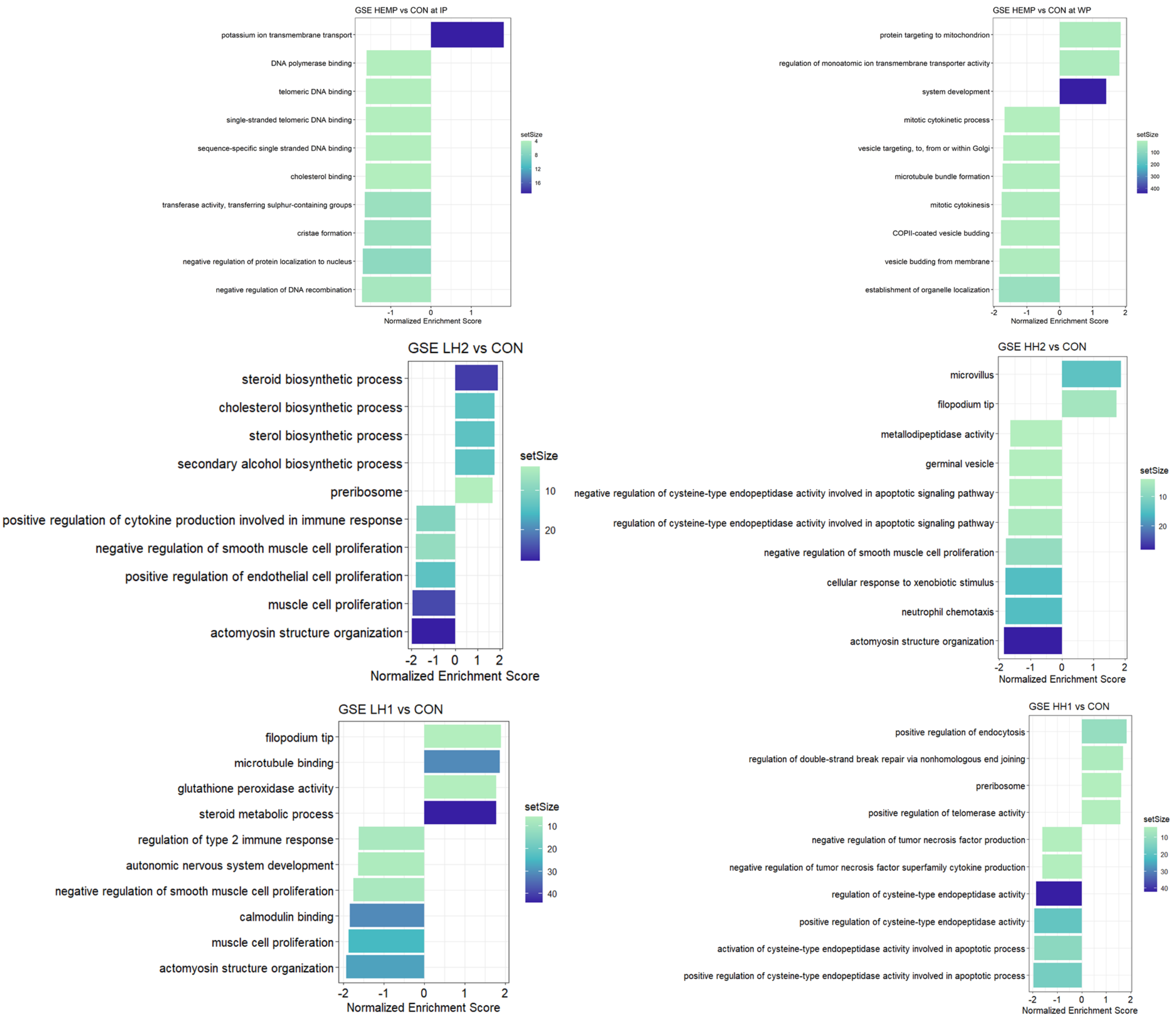

Figure S2. Top-10 functional annotations of differentially expressed genes of dairy cows and lambs fed spent hemp biomass, analyzed using gene-set enrichment (GSE) analysis in ClusterProfiler R package.

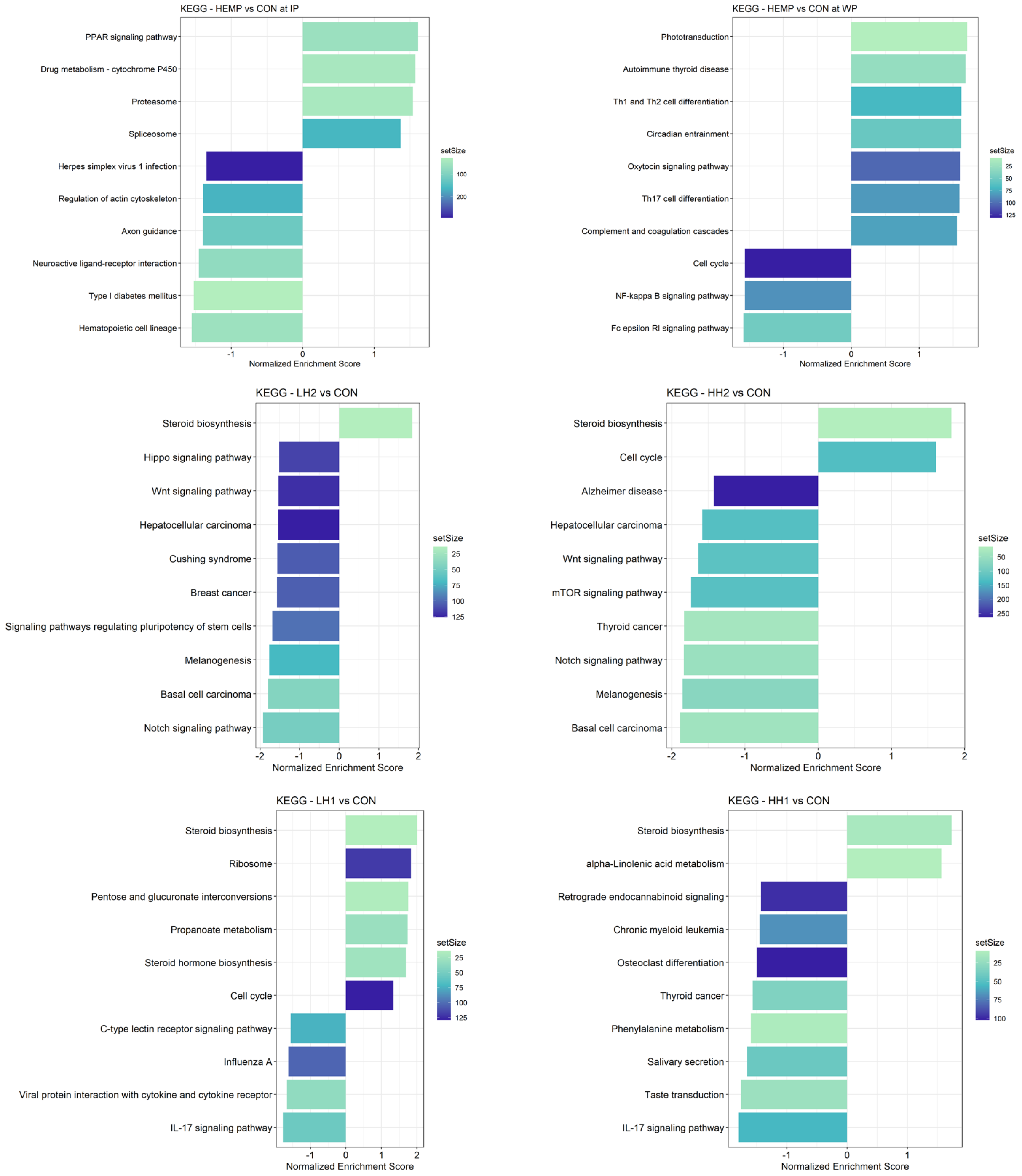

Figure S3. GSEA most enriched KEGG pathways in dairy cows and lambs fed spent hemp biomass

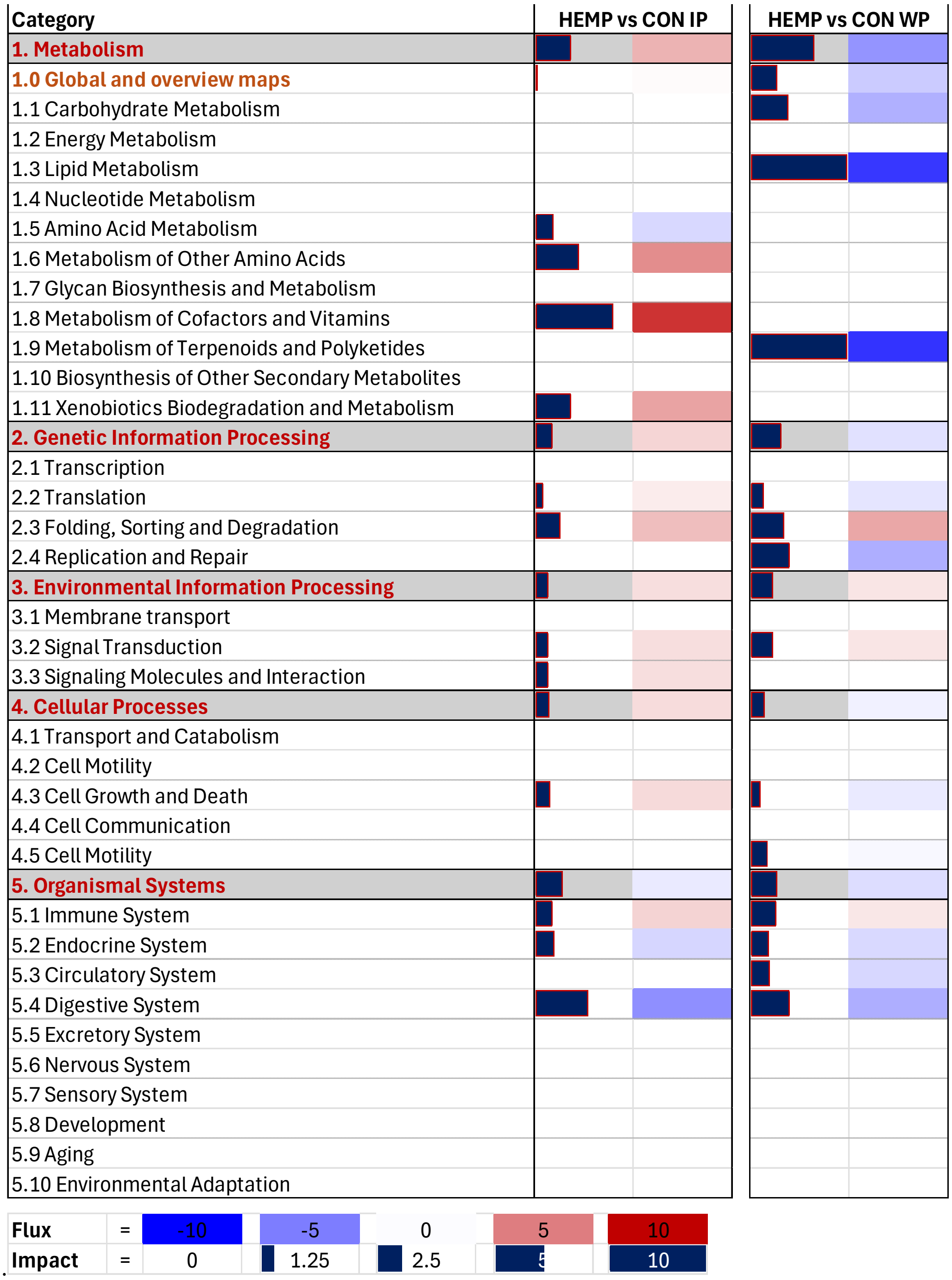

Figure S4. Dynamic Impact Approach summary of Kyoto Encyclopedia of Genes and Genomes pathways of liver transcriptomic of dairy cows fed spent hemp biomass (SHB) or a control diet (CON) during the intervention (IP) or withdrawal (WP) period.

Dairy cows – HEMP vs. CON at IP

10 most overall impacted Terms	Impact	Flux	Rank
Nicotinate and nicotinamide metabolism			1
Protein digestion and absorption			2
Glutathione metabolism			3
Drug metabolism - cytochrome P450			4
Metabolism of xenobiotics by cytochrome P450			5
Chemical carcinogenesis			6
Salmonella infection			7
Ubiquitin mediated proteolysis			8
Platinum drug resistance			9
Toll-like receptor signaling pathway			10

Dairy cows – HEMP vs. CON at WP

10 most overall impacted Terms	Impact	Flux	Rank
Fatty acid biosynthesis			1
Terpenoid backbone biosynthesis			2
Sphingolipid metabolism			3
Non-homologous end-joining			4
Fructose and mannose metabolism			5
Fatty acid metabolism			6
AMPK signaling pathway			7
Bile secretion			8
Gastric acid secretion			9
Complement and coagulation cascades			10

Impact	0	5	10
Flux	-10	0	10

Lambs – LH1 vs. CON

10 most overall impacted Terms	Impact	Flux	Rank
Valine, leucine and isoleucine biosynthesis			1
Phenylalanine, tyrosine and tryptophan biosynthesis			2
Taurine and hypotaurine metabolism			3
Biosynthesis of unsaturated fatty acids			4
Folate biosynthesis			5
Sulfur metabolism			6
Vitamin B6 metabolism			7
Steroid biosynthesis			8
Selenoamino acid metabolism			9
RIG-I-like receptor signaling pathway			10

Lambs – HH1 vs. CON

10 most overall impacted Terms	Impact	Flux	Rank
Phenylalanine metabolism			1
Tyrosine metabolism			2
Folate biosynthesis			3
Oxidative phosphorylation			4
Parkinson's disease			5
Non-alcoholic fatty liver disease (NAFLD)			6
Sulfur metabolism			7
Alzheimer's disease			8
Huntington's disease			9
Renin-angiotensin system			10

Lambs – LH2 vs. CON

10 most overall impacted Terms	Impact	Flux	Rank
Basal cell carcinoma			1
Notch signaling pathway			2
Melanogenesis			3
Wnt signaling pathway			4
Hippo signaling pathway			5
Signaling pathways regulating pluripotency of stem cells			6
Taurine and hypotaurine metabolism			7
mTOR signaling pathway			8
Steroid biosynthesis			9
Breast cancer			10

Lambs – HH2 vs. CON

10 most overall impacted Terms	Impact	Flux	Rank
Basal cell carcinoma			1
Notch signaling pathway			2
Melanogenesis			3
Phenylalanine, tyrosine and tryptophan biosynthesis			4
Signaling pathways regulating pluripotency of stem cells			5
Dorso-ventral axis formation			6
Breast cancer			7
Hippo signaling pathway			8
Wnt signaling pathway			9
Steroid biosynthesis			10

Impact	0	50	100
Flux	-100	0	100

Figure S5. Top-10 impacted pathways of each comparison as highlighted by dynamic impact approach

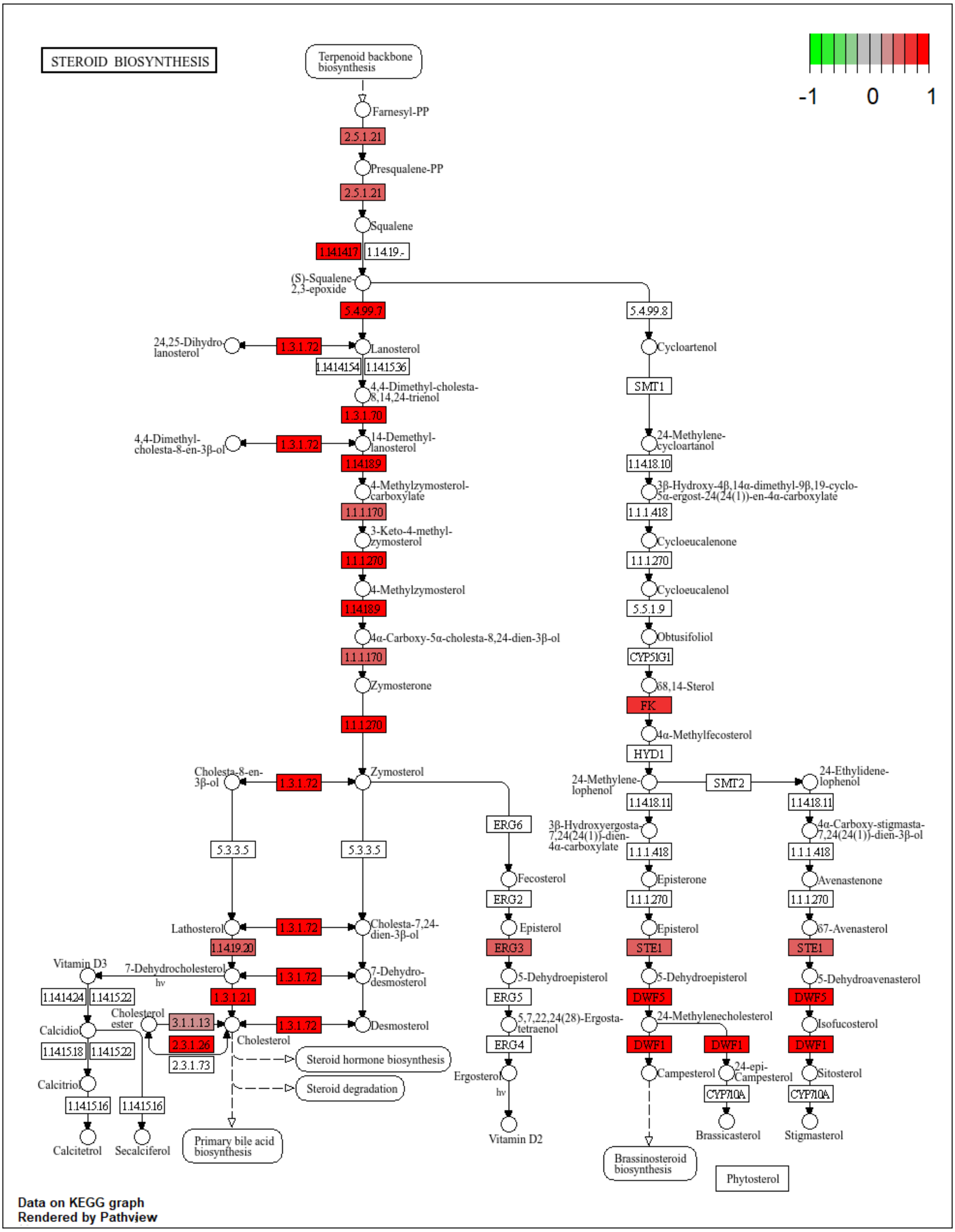

Figure S6. Steroid biosynthesis pathway affected by feeding spent hemp biomass in lambs (HH2 group)

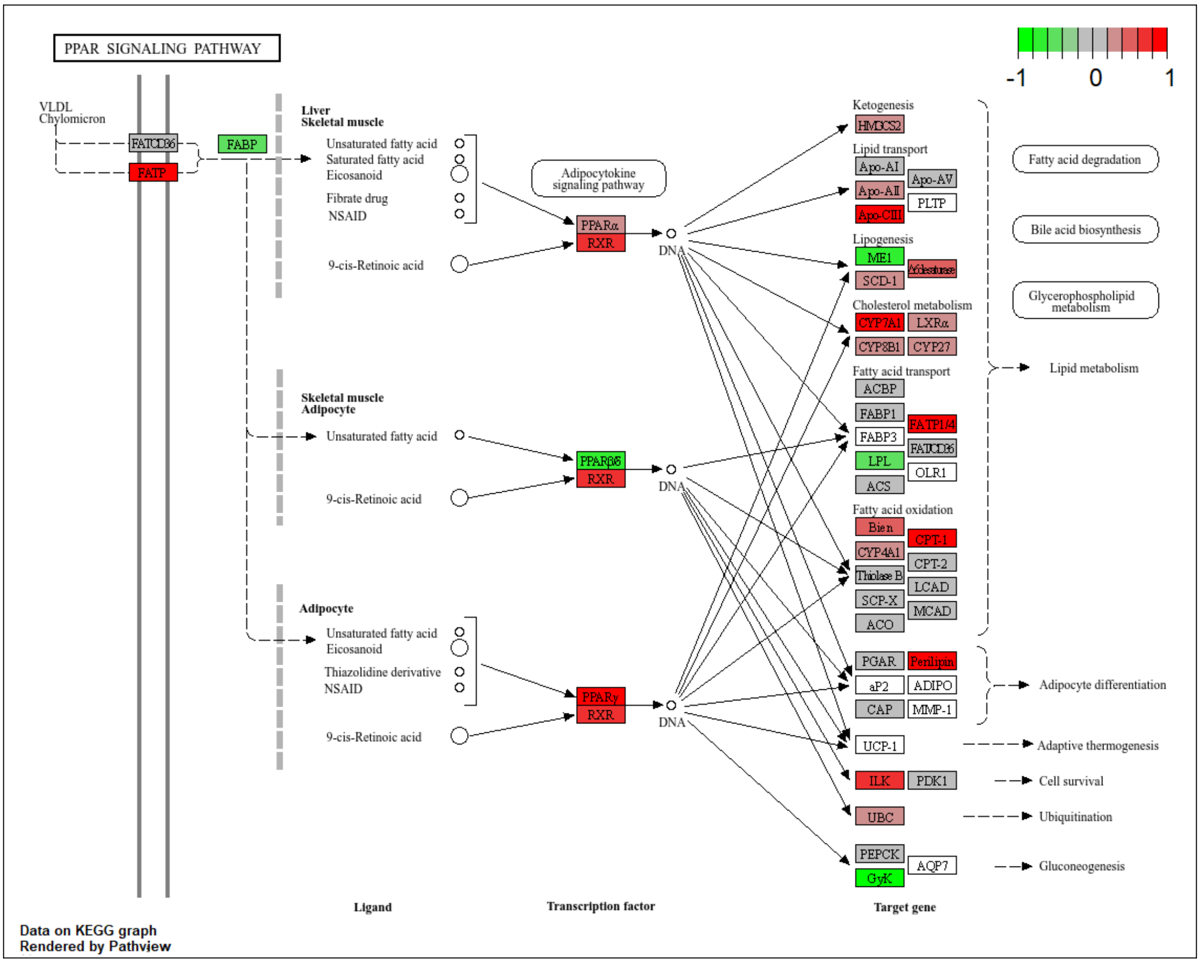

Figure S7. Effect on PPAR signaling pathway by feeding spent hemp biomass to dairy cows

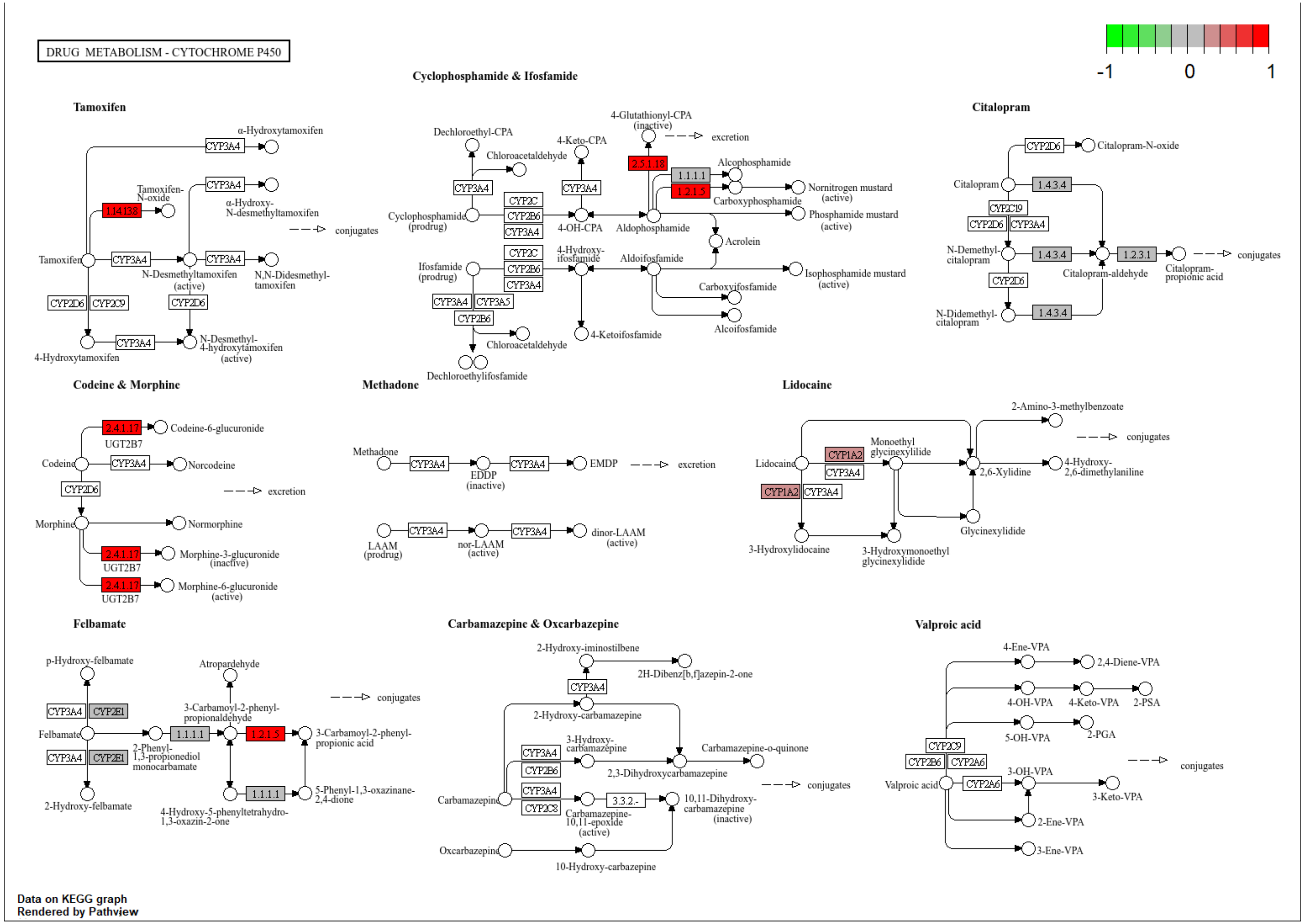

Figure S7. Modulation of drug metabolism related to cytochrome P450 pathway due to dietary spent hemp biomass fed to dairy cows

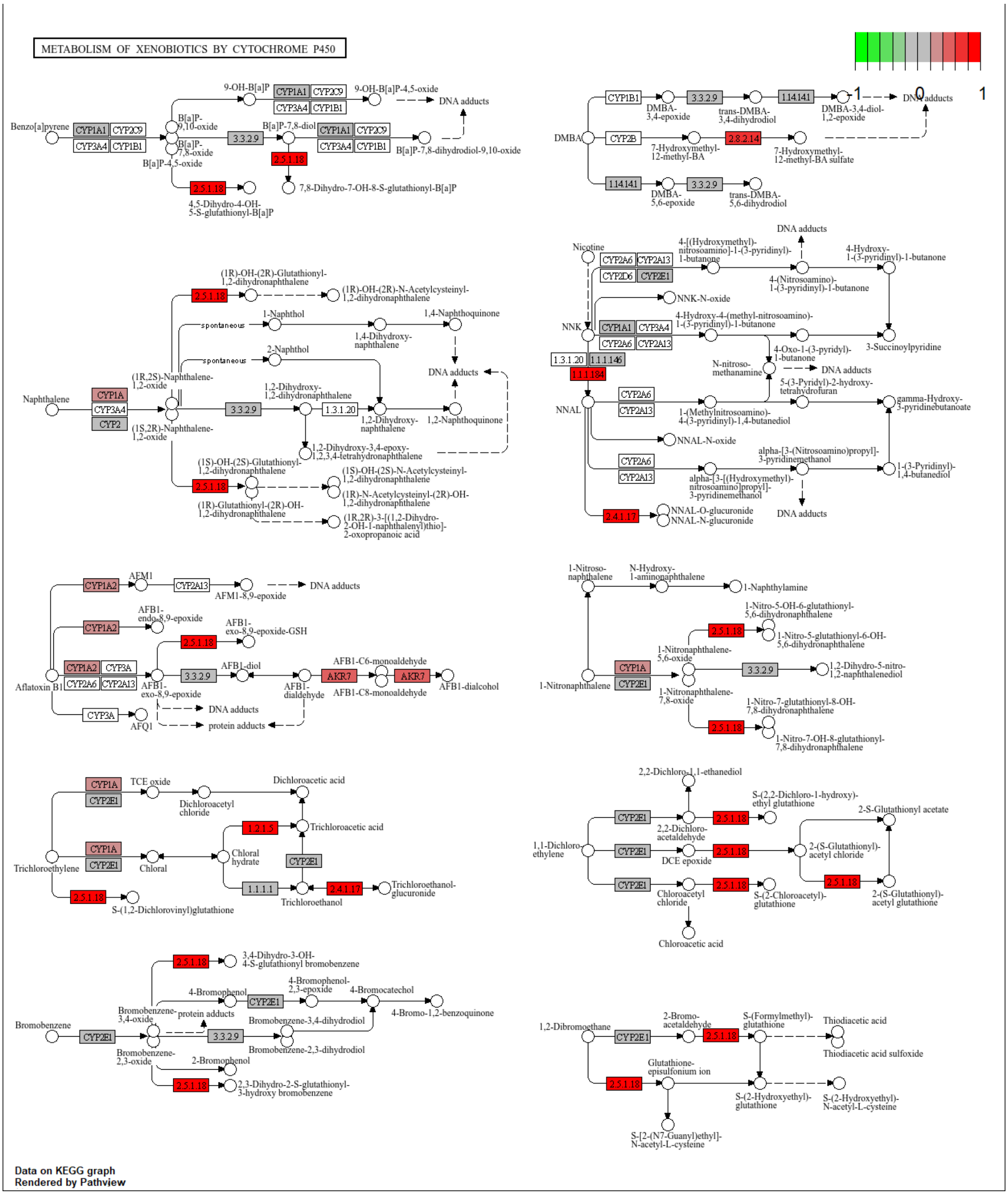

Figure S8. Modulation of metabolism of xenobiotics pathway by cytochrome P450 due to dietary spent hemp biomass fed to dairy cows

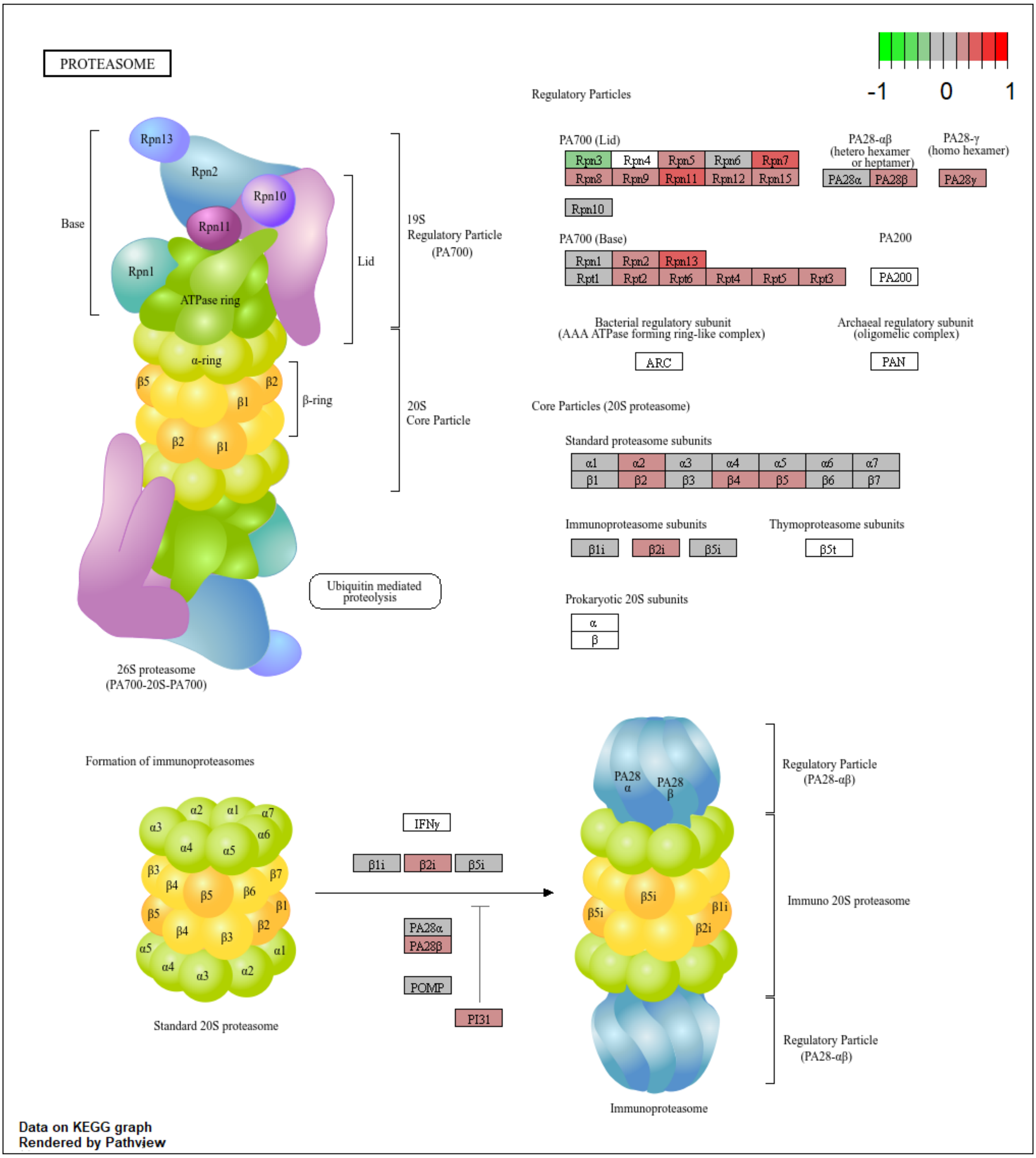

Figure S9. Peroxisome pathway affected by feeding spent hemp biomass in dairy cows
